# Treatment and genetic analysis of multiple supernumerary and impacted teeth in an adolescent patient

**DOI:** 10.1186/s12903-024-04573-3

**Published:** 2024-07-14

**Authors:** Panjun Pu, Yuxia Hou, Qing Zhang, Xiaoyi Hu, Yi Ding, Peizeng Jia, Huaxiang Zhao

**Affiliations:** 1 Key Laboratory of Shaanxi Province for Craniofacial Precision Medicine Research, College of Stomatology, Xi’an Jiaotong University, No. 98, Xiwu Road, Xincheng District, Xi’an, Shaanxi 710004 PR China; 2 Department of Orthodontics, College of Stomatology, Xi’an Jiaotong University, Xi’an, Shaanxi PR China; 3 Department of Cranio-Maxillofacial Trauma and Plastic Surgery, College of Stomatology, Xi’an Jiaotong University, Xi’an, Shaanxi PR China; 4 Department of Physiology and Pathophysiology, School of Basic Medical Sciences, Xi’an Jiaotong University, Xi’an, Shaanxi PR China; 5 Department of Orthodontics, Peking University School and Hospital of Stomatology, No. 22 Zhongguancun South Ave, Beijing, 100081 PR China

**Keywords:** Supernumerary tooth, Impacted tooth, Whole-exome sequencing, Genetic analysis, *TCF7L2*

## Abstract

**Background:**

Multiple supernumerary teeth, combined with numerous impacted teeth, can lead to various malocclusions, posing significant treatment challenges. While certain genes associated with syndromic cases of multiple supernumerary and impacted teeth have been identified, the etiologies of non-syndromic cases still largely remain elusive.

**Case presentation:**

Here, we report a treatment of a 12-year-old boy who presented with 10 supernumerary teeth and 6 impacted teeth, accompanied by a genetic analysis to explore the underlying etiology. During the treatment, fifteen teeth were extracted, and various skilled techniques, including the closed-eruption technique and the application of by-pass arches, were utilized. Post-treatment, traction was successful for all the impacted teeth, without any tooth mobility or reduction in gingival height. Space closure, well-aligned teeth, and excellent functional occlusion were achieved. Furthermore, comprehensive genetic analysis was conducted through whole-exome sequencing on the patient and his parents, which revealed a potential link between the patient’s numerous supernumerary teeth and abnormal mineralization. Notably, the p.Ser496Pro variant in the *TCF7L2* gene was identified as a potential candidate variant in this patient.

**Conclusions:**

Overall, our findings not only report the treatment of a rare case involving multiple supernumerary and impacted teeth but also offer valuable insights into the molecular basis of supernumerary teeth.

**Supplementary Information:**

The online version contains supplementary material available at 10.1186/s12903-024-04573-3.

## Background

Supernumerary tooth is one of the most common dental anomalies, with a prevalence ranging between 1.11% and 14.04%, depending on age, sex, and geographical region [1, 2]. Compared to the solitary supernumerary tooth, the occurrence of more than five supernumerary teeth is considerably rare [3].

Supernumerary teeth, especially when multiple supernumerary teeth are present, can lead to a variety of malocclusions, including delayed eruption of permanent teeth, arch crowding, and impacted teeth [4, 5]. The management of impacted teeth resulting from multiple supernumerary teeth poses significant challenges. This process entails meticulous strategizing regarding the identification of teeth suitable for traction and those that should be extracted. A prudent surgical technique is imperative during tooth extraction to avert potential damage to both teeth and periodontal tissues, as well as careful consideration of the direction and anchorage of the traction [6].

Multiple supernumerary teeth combined with multiple impacted teeth commonly manifest as indicators of certain craniofacial syndromes, including cleidocranial dysplasia (CCD), tricho-rhino-phalangeal syndrome, and Robinow syndrome [7–9]. These syndromes typically result from Mendelian disorders arising from single-gene variants [10, 11]. However, the etiology of non-syndromic multiple supernumerary and impacted teeth has rarely been reported [12, 13].

In the present study, we presented the phenotype and subsequent orthodontic treatment of a 12-year-old boy who exhibited 10 supernumerary teeth and 6 impacted teeth and underwent genetic analysis to investigate the underlying etiology.

## Case presentation

### Medical history, symptoms, and diagnosis

A 12-year-old boy came to the Department of Orthodontics with the chief complaint of multiple permanent teeth failing to erupt after the natural loss of his deciduous teeth. To investigate any underlying systemic developmental issues, a thorough medical history was taken. Notably, there was no indication of any phenotypic abnormalities or a history of motor developmental delays, hyperactivity, or bipolar disorder in this patient. It’s worth mentioning that the patient exhibited proficiency in both mathematics and sports at school. A thorough inquiry into the family history indicated that the parents did not demonstrate a similar phenotype of permanent teeth not erupting.

**Fig. 1:**
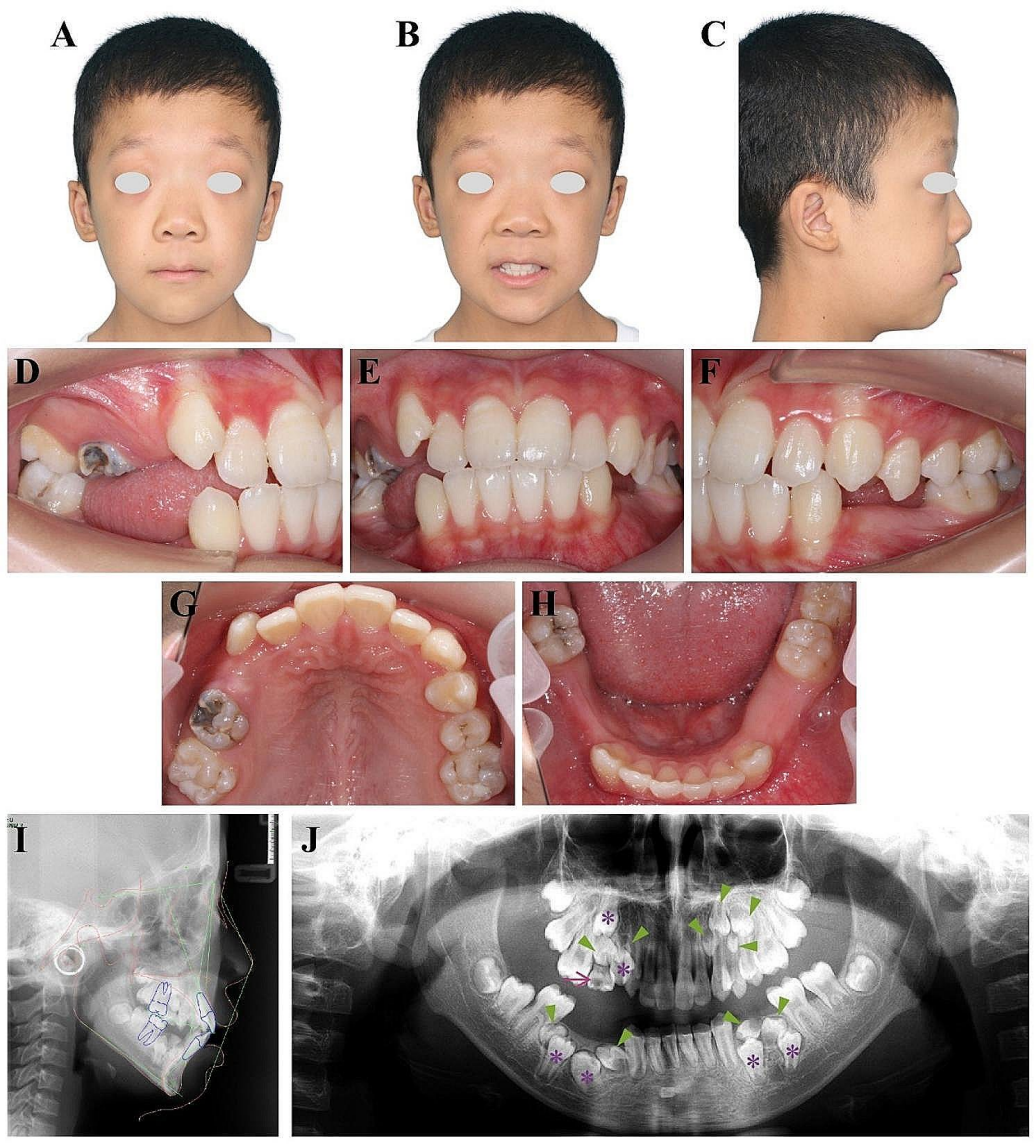
Pretreatment examinations of the patient with multiple supernumerary and impacted teeth. **(A-C)** Pretreatment facial photographs. **(D-H)** Pretreatment intraoral photographs. **(I)** Pretreatment lateral cephalogram. **(J)** Pretreatment panoramic radiograph. Impacted permanent teeth are denoted by purple asterisks. Supernumerary teeth are indicated by green arrowheads, while the retained deciduous tooth is indicated by the purple arrow

The general physical examination showed that the patient’s height fell within the normal range, while his weight and physique appeared to be somewhat thin. Facial examination revealed several distinctive features, including a broad forehead, flattened nasal bridge, and increased eye distance. The boy showed a slightly convex facial profile, with a minor protrusion of both the upper and lower lips (Fig. 1A-C). The absence of premolars in the upper right and lower arch resulted in a noticeable 6 mm space in the upper arch and a substantial 27 mm space in the lower arch. The upper right second deciduous molar was retained, and the upper right canine displayed a slightly elevated position. On the left side, the boy presented a Class I canine and molar relationship, while on the right side, a Class II canine and molar relationship was observed. The maxillary dental midline was aligned with the facial midline, but a slight deviation to the right was observed in the mandibular dental midline (Fig. 1D-H).

Next, imaging examinations, including lateral cephalography and panoramic radiography, were performed to evaluate his dental structure. The pretreatment lateral cephalometric assessment indicated a mild skeletal Class II relationship and a high mandibular plane. Both the maxillary and mandibular incisors exhibited upright positions within the normal range (Fig. 1I and Table 1). The pretreatment panoramic radiography revealed the presence of numerous supernumerary teeth and multiple impacted teeth in the patient. In further detail, we identified two supernumerary teeth in the upper right arch, four in the upper left arch, and two on each side of the lower arch (indicated by green arrowheads in Fig. 1J). Furthermore, the upper right first and second premolars, along with all lower first and second premolars, were impacted within the maxillary and mandibular bones (marked by purple asterisks in Fig. 1J). Overall, this patient exhibited a total of 10 supernumerary teeth and 6 impacted teeth.

**Table 1:** Cephalometric measurements

Measurement	Norm	Pretreatment	Posttreatment
SNA°	82.8 ± 4.0	78.1	79.1
SNB°	80.1 ± 3.9	73.5	73.7
ANB°	2.7 ± 2.0	4.5	5.3
FMA°	31.3 ± 5.0	43.7	42.9
SN-GoGn°	32.5 ± 5.2	44.0	43.5
U1-NA°	22.8 ± 5.7	23.7	15.3
U1-SN°	105.7 ± 6.3	101.7	94.4
L1-NB°	30.3 ± 5.8	35.7	35.5
IMPA (L1-MP)°	92.6 ± 7.0	94.9	95.1

Additionally, we performed a chest radiograph to assess the condition of the sternum and clavicle, indicating no abnormalities in these areas (Supplemental Fig. 1).

Based on these examinations, the patient was diagnosed with multiple supernumerary teeth, multiple impacted teeth and a high mandibular plane.

### Treatment plans

Supernumerary teeth needed to be extracted to prevent potential complications. Subsequently, three treatment alternatives were considered for the boy: (1) The first option involved traction of all the impacted teeth, closing the spaces in both arches, and achieving functional occlusion. This approach required an extended treatment duration and posed a risk of traction failure and tooth mobility after extraction. (2) The second option included extracting selected impacted teeth presenting difficulties in traction or with suboptimal root shape, combined with the traction of other impacted teeth. Similar to the first option, this approach also exhibited the challenges associated with traction of the impacted teeth. Additionally, due to the extraction of more teeth than in the first option, achieving space closure was possibly more complex. (3) The third option entailed the extraction of all impacted teeth, with dentures employed for space restoration. This option required no orthodontic treatment, but the effectiveness of dentures, especially the use of removable dentures during the patient’s pre-adult years, was inferior to that of natural teeth in terms of occlusal function.

After presenting the treatment options along with their risks and benefits, the boy and his parents opted for the second option. The third option remained as an alternative in case space closure proved unattainable.

### Treatment methodology and progress

**Fig. 2:**
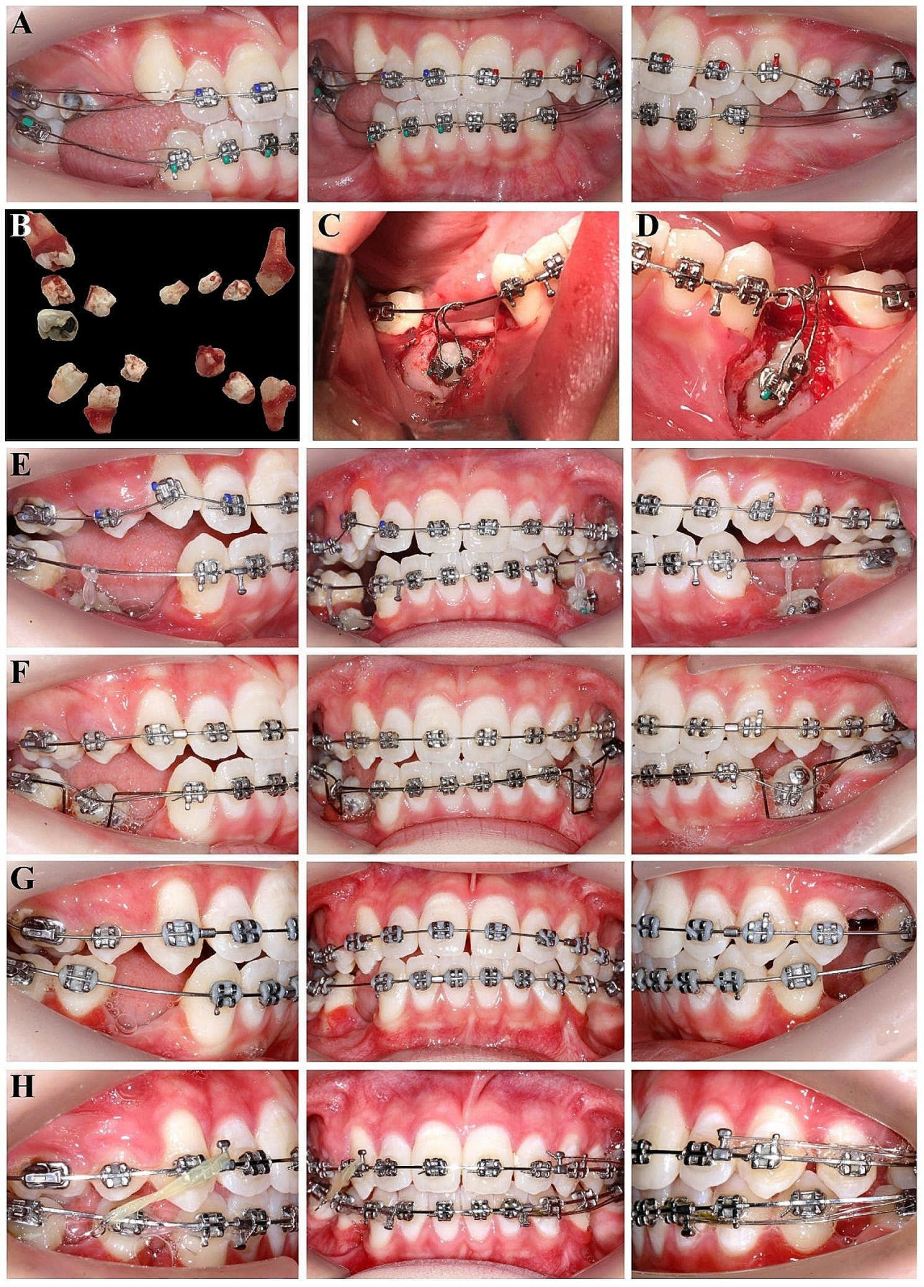
Progress intraoral photos during the treatment. **(A)** Initial treatment phase: bonding a fixed straight wire appliance (SWA) on all teeth, accompanied by the placement of 0.012-inch nickel-titanium (NiTi) archwires. Notably, the intentional misalignment of the upper right canine was performed at this stage to prevent anterior teeth from inclining labially. **(B)** 4-month stage: teeth extraction under general anesthesia, with concurrent extraction of the upper right second premolar, the lower right first premolar, the lower left second premolar, the retained upper right second deciduous molar and all supernumerary teeth. **(C and D)** Closed eruption technique: bonding lingual buttons and brackets on the impacted **(C)** lower right second premolar and **(D)** lower left first premolar, utilizing a closed-eruption approach. Remarkably, each impacted premolar was affixed with both a lingual button and a bracket to ensure steadfast bonding throughout subsequent tooth traction. **(E)** 7-month stage: spontaneous eruption of the impacted upper right first premolar and utilization of a 0.012-inch NiTi wire to align the upper arch. Elastic chains employed to guide the impacted lower premolars towards a 0.016 × 0.022-inch stainless-steel (SS) archwire in the lower arch. **(F)** 9-month stage: adoption of a by-pass arch technique to align the lower premolars. Primary archwire: 0.016-inch Australian wire; auxiliary archwire: 0.012-inch NiTi wire. **(G)** 14-month stage: Successful traction of all impacted premolars, achieving alignment in both the upper and lower dentition. Owing to a Class I canine relationship bilaterally and normal anterior overjet, extraction of the upper left second premolar was undertaken. **(H)** Space closure: transition to 0.018 × 0.025-inch SS archwires for space closure employing sliding mechanics. Introduction of Class II elastics (1/4 or 3/16, 3.5 oz) to mesialize the lower right first molar

The commencement of treatment involved bonding a fixed straight wire appliance (SWA, Shinye *lnc.*, China) on all teeth and placing 0.012-inch nickel-titanium (NiTi) archwires. During this phase, we kept the upper right canine in a non-aligned position, to forestall labial inclination of the anterior teeth (Fig. 2A). The sequential placement of the NiTi archwires achieved alignment and levelling. Following the placement of 0.016 × 0.022-inch stainless-steel (SS) archwires at 4 months, the patient underwent extraction surgery under general anaesthesia, with concurrent extraction of the upper right second premolar, lower right first premolar, lower left second premolar, retained upper right second deciduous molar, and all the supernumerary teeth (all extracted teeth shown in Fig. 2B). During the extraction surgery, lingual buttons and brackets were affixed to the impacted premolars using a closed eruption technique. A lingual button and bracket were simultaneously bonded to each impacted premolar to avoid the need for a second surgery due to potential loss of the traction attachments (Fig. 2C and D).

By 7 months, the impacted upper right first premolar spontaneously erupted, and a 0.012-inch NiTi archwire was employed to achieve alignment of the upper arch. In the lower arch, elastic chains were employed to guide the impacted lower premolars onto a 0.016 × 0.022-inch SS archwire (Fig. 2E). At 9 months, a by-pass arch technique was executed to facilitate alignment of the lower premolars. This technique involved employing a 0.016-inch Australian wire as the primary archwire, and a 0.012-inch NiTi wire as the auxiliary archwire (Fig. 2F).

By 14 months, successful traction of all impacted premolars was realized. Synchronously, alignment of both upper and lower dental arches was accomplished. Owing to a Class I canine relationship bilaterally and a normal anterior overjet, extraction of the upper left second premolar was undertaken (Fig. 2G). We transitioned to 0.018 × 0.025-inch SS archwires for space closure with the application of sliding mechanics. Class II elastics (1/4 or 3/16, 3.5 oz) were applied to mesialize the mandibular right first molar, thereby achieving a Class I molar relationship on the right side (Fig. 2H).

After debonding of the brackets, fixed retainers were bonded in both the upper and lower arches.

### Treatment results and the emergence of a new supernumerary tooth during orthodontic treatment

**Fig. 3:**
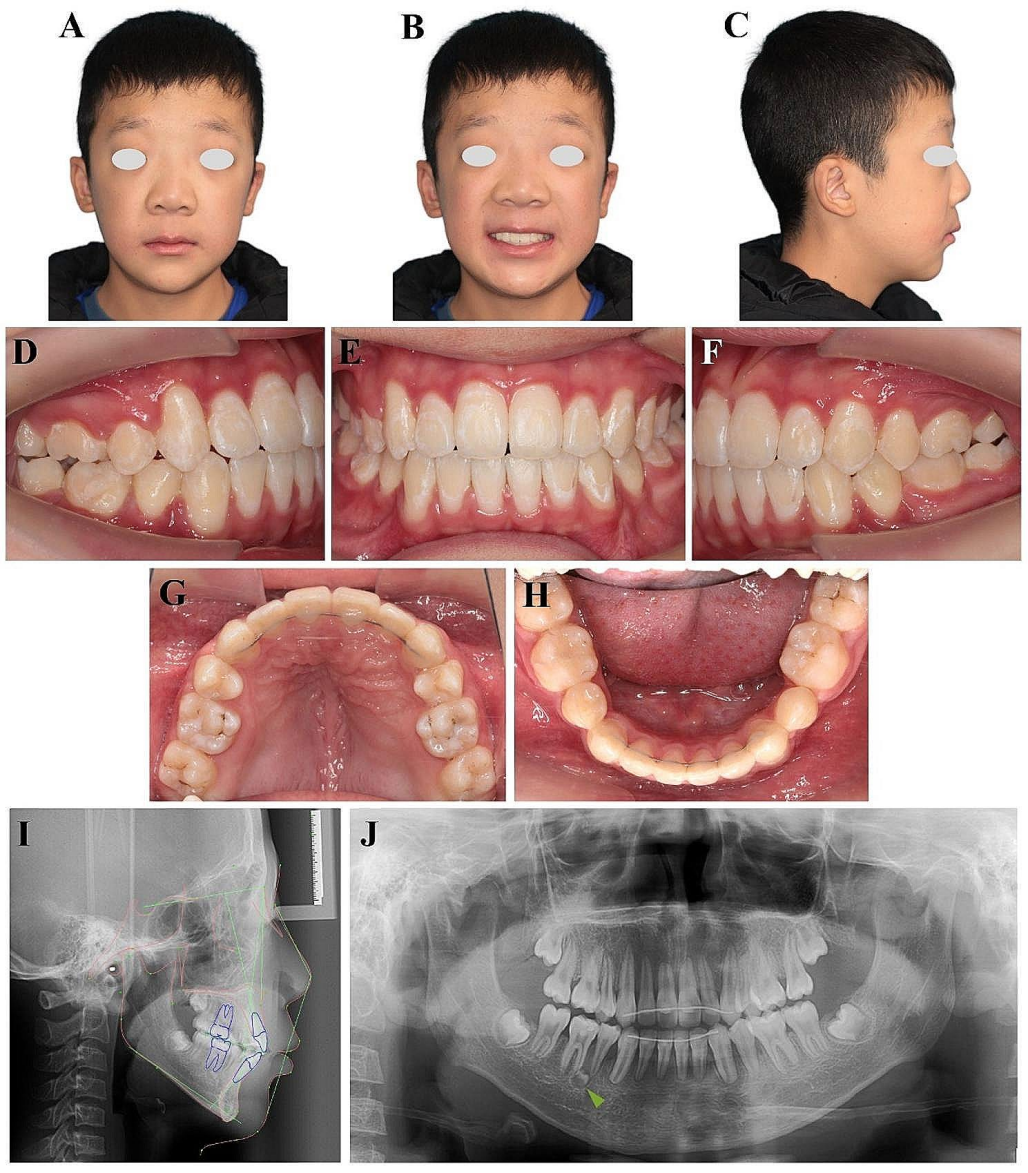
Posttreatment examinations of this patient. **(A-C)** Posttreatment facial photographs. **(D-H)** Posttreatment intraoral photographs. **(I)** Posttreatment lateral cephalogram. **(J)** Posttreatment panoramic radiograph. Notably, a new supernumerary tooth (indicated by the green arrowhead) emerged post to the teeth extraction surgery

The overall duration of active treatment was 39 months. No discernible changes were observed upon evaluation of the facial photographs (Fig. 3A-C). Despite not utilizing micro-implants or extraoral anchorages, both the upper and lower arch midlines achieved alignment that corresponded with the facial midline (Fig. 3B, D-H). Intraoral examinations showed harmonious maxillary and mandibular arches, well-aligned teeth and successful closure of spaces (Fig. 3G and H). The patient presented a bilateral Class I canine and molar relationship, accompanied by a satisfactory overjet/overbite of anterior teeth, as well as excellent functional occlusion of posterior teeth (Fig. 3D-H). All the impacted teeth that underwent traction displayed vitality without any tooth mobility, and the gingival height was maintained (Fig. 3D-H).

Assessment of cephalometric measurements and superimpositions revealed notable growth in the maxillary and mandibular bones, along with developmental changes in facial soft tissues. In addition, subtle retraction of the maxillary and mandibular incisors was observed (Fig. 3I, Supplemental Fig. 2, and Table 1). Panoramic radiographs demonstrated successful traction of all impacted teeth, accompanied by acceptable root parallelism (Fig. 3J). While the roots presented a subtle rounding and blunting, the crown-root ratio remained within the acceptable range (Fig. 3J). Radiographic imaging also revealed the presence of four third molars, which were recommended for extraction (Fig. 3J). Surprisingly, despite the absence of supernumerary teeth mesial to the root of the lower right first molar after tooth extraction surgery (Supplemental Fig. 3), a new supernumerary tooth emerged after orthodontic treatment (indicated by green arrowheads in Fig. 3J), suggesting aberrant odontogenic activity in this patient.

The patient and his parents were satisfied with the functional and aesthetic outcomes of treatment. It was recommended to extract the new supernumerary tooth mesially to the root of the lower right first molar. However, since this tooth was deeply embedded in the mandible and there was no apparent damage or symptoms, the patient and his parents chose not to undergo extraction surgery. A stable occlusal relationship was maintained at the 10-month follow-up (Supplemental Fig. 4).

### Genetic analysis indicating that the patient’s supernumerary teeth were associated with abnormal mineralization and the TCF7L2 p.Ser496Pro variant

The presence of numerous supernumerary teeth, especially the emergence of a new supernumerary tooth after tooth extraction surgery, led us to strongly suspect an abnormal regulation of certain genes in this patient. Despite the presence of several distinct facial and dental characteristics, the absence of abnormal phenotypes in other organs and the lack of neurodevelopmental abnormalities rendered it difficult to conclusively associate the phenotype of numerous supernumerary/impacted teeth with syndromes as the underlying cause. Therefore, we conducted whole-exome sequencing (WES; BGISEQ-500 platform, BGI *Inc.*, China) on both the patient and his parents.

**Fig. 4:**
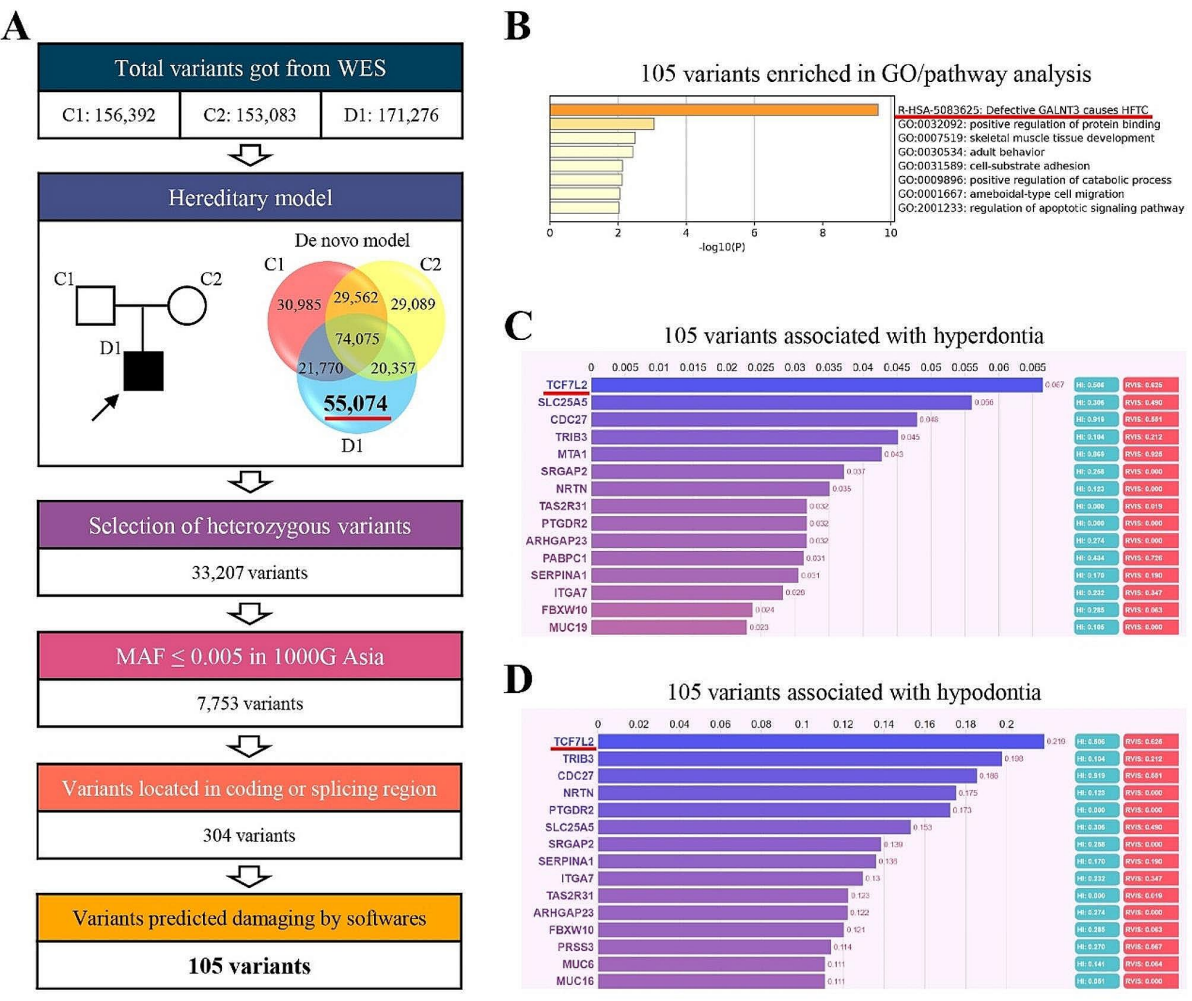
Genetic analysis of the patient with multiple supernumerary teeth. **(A)** Screening process to identify candidate variants in the patient with multiple supernumerary teeth. We performed whole-exome sequencing (WES) on the patient (D1) and his unaffected parents (C1 and C2). Next, we employed a de novo hereditary model to narrow down the list of candidate variants, yielding a total of 55,074 variants in this patient (marked with the red underline). To further refine the candidate variants, we focused on heterozygous variants and those with a low frequency (MAF < 5‰) in the 1000G_EAS database. Specifically, variants causing amino acid changes (missense, nonsense, insertion, deletion, etc.) or splicing variants were included for the subsequent analysis. Finally, we evaluated the potential effects of these variants by softwares and identified 105 candidate variants. **(B)** Major enriched Gene Ontology (GO)/pathway terms among the 105 candidate variants. Notably, the most prominently enriched term (indicated by the red underline) is linked to mineralization processes (calcinosis), suggesting that the presence of multiple supernumerary teeth in the patient might be attributed to abnormal mineralization caused by the identified candidate variants. **(C and D)** Analysis of 105 candidate variants related to **(C)** hyperdontia and **(D)** hypodontia using Phenolyzer software. Of significant note, *TCF7L2* (also known as *TCF4*, indicated by the red underline), a gene linked to tooth development, emerged as the top-ranking candidate, suggesting that *TCF7L2* (p.Ser496Pro) might potentially be the crucial candidate variant of this patient

Employing a similar screening strategy to previous studies [14], we identified a total of 105 candidate variants (Fig. 4A). The GO/pathway analysis indicated that the most significantly enriched term was linked to mineralization processes (calcinosis), implying that the patient’s multiple supernumerary teeth might result from abnormal mineralization triggered by the identified candidate variants (Fig. 4B).

Subsequently, two hypotheses were formulated: the presence of gain-of-function odontogenic variants and/or loss-of-function anti-odontogenic variants. To investigate these hypotheses, we utilized the Phenolyzer software [15] to conduct a variant-phenotype analysis. Remarkably, *TCF7L2* and its variant (c.1486T > C/p.Ser496Pro), a gene associated with tooth development, emerged as the foremost candidate (Fig. 4C and D), suggesting that this variant could be the pivotal candidate variant contributing to numerous supernumerary teeth in the patient.

## Discussion

Supernumerary teeth, particularly when present in large numbers, have been reported as contributors to a range of dental complications, including delayed or failed eruption of permanent teeth, severe displacement of teeth, and root resorption of neighboring teeth, consequently affecting oral function [16]. Moreover, certain pathological complications can arise due to the presence of supernumerary teeth, including the formation of odontogenic tumors or cysts [17]. In this study, the presence of misaligned supernumerary teeth appeared to be the underlying cause for the prevalence of impacted teeth. For instance, the supernumerary tooth positioned coronal to the upper right second premolar likely obstructed the normal eruption path of this tooth, leading to its impaction (Fig. 1J). A similar scenario was evident in the lower dental arch (Fig. 1J), highlighting the necessity for early identification of supernumerary teeth to prevent the potential impaction of teeth due to the presence of supernumerary teeth [18, 19].

A well-thought-out design is pivotal for the successful management of cases with multiple impacted teeth. In this patient, the upper right second premolar was positioned at an excessive vertical height, prompting the decision to extract this tooth rather than pursue traction. As for the lower impacted premolars, the lower right first premolar exhibited unfavorable morphology, while the lower left second premolar was obstructed by the first molar, therefore, both premolars were selected for extraction.

Previous studies have strongly recommended the utilization of the closed-eruption technique for impacted teeth situated at high positions within the alveolar bone [20]. Compared to the open-eruption technique, while the closed-eruption technique does require a longer traction duration, it yields superior aesthetic outcomes in terms of crown length and gingival height [20]. However, potential concerns related to attachment loss during the traction process and the need for a second surgery have been associated with the implementation of the closed-eruption approach. To circumvent these potential issues, a lingual button and bracket were simultaneously bonded to each impacted premolar (Fig. 2C and D). In addition, a by-pass arch technique was employed during the alignment of the impacted premolars (Fig. 2F). This approach enabled the impacted teeth to move without any interference from the main archwire, thereby accelerating the alignment process.

The patient presented with distinctive facial features and numerous supernumerary/impacted teeth, prompting suspicions of some potential syndromes, particularly CCD [21–23]. In light of this suspicion, chest radiography and genetic testing were conducted. The chest radiograph did not reveal any signs of clavicular dysplasia (Supplemental Fig. 1), and the genetic analysis did not identify any rare variants in the *RUNX2* gene (the causal gene of CCD [24], data not shown). These findings significantly diminished the likelihood of CCD in this patient. Previous studies have suggested that the occurrence of supernumerary teeth is linked to the dichotomy of tooth buds, heightened activity of the dental lamina and genetic factors [10, 25]. Talaat et al. have demonstrated an association between supernumerary teeth and dysregulation of critical signaling pathways such as WNT and SHH [26, 27]. In addition, a missense variant in *PDGFRB* [12] and a heterozygous variant in *FER1L6* [28] have also been reported to be associated with non-syndromic supernumerary teeth. However, the precise etiology of non-syndromic supernumerary teeth remains elusive. Therefore, we performed WES on this family. Bioinformatics analysis uncovered a possible link between the patient’s multiple supernumerary teeth and abnormal mineralization, with a notable candidate rare variant identified in *TCF7L2* (p.Ser496Pro) (Fig. 4). TCF7L2, also known as TCF4, plays a pivotal role as a transcriptional effector in the WNT/β-catenin signaling pathway, which has suggested involvement in tooth development [29–31]. The identified rare variant, p.Ser496Pro, could potentially be a gain-of-function variant that augments the ability of tooth formation, thereby contributing to numerous supernumerary teeth in the patient. Further studies are required to ascertain the pathogenicity of this *TCF7L2* variant, shedding light on the molecular basis of non-syndromic supernumerary teeth.

## Conclusions

We made two main findings in this report. First, we reported the treatment of a rare case involving multiple supernumerary and impacted teeth. Second, the genetic investigation indicated that the patient’s multiple supernumerary teeth might be linked to abnormal mineralization, and the *TCF7L2* p.Ser496Pro variant was identified as a potential candidate variant in this patient. Therefore, our work offers valuable insights into the molecular basis of supernumerary teeth.

### Electronic supplementary material

Below is the link to the electronic supplementary material.

Supplementary Material 1

## Data Availability

The datasets generated and/or analysed during the current study are available in the Genome Variation Map (GVM) in National Genomics Data Center, Beijing Institute of Genomics, Chinese Academy of Sciences and China National Center for Bioinformation, under accession number GVM000787.

## Abbreviations

CCD: Cleidocranial dysplasia
NiTi: Nickel-titanium
SS: Stainless-steel
SWA: Straight wire appliance
WES: Whole-exome sequencing
