# Supplementary material for: Treatment and genetic analysis of multiple supernumerary and impacted teeth in an adolescent patient
Source: BMC Oral Health. 2024 Jul 14;24:790. doi: 10.1186/s12903-024-04573-3 (PMC11246580; doi:10.1186/s12903-024-04573-3)
Supplement: Supplementary file 1 — Supplementary Material 1 [file 12903_2024_4573_MOESM1_ESM.pdf]

Supplemental figures for

Treatment and genetic analysis of multiple supernumerary and impacted teeth in an adolescent patient

Panjun Pu[1, 2, †], Yuxia Hou[1, 2, †], Qing Zhang[1, 2], Xiaoyi Hu[1, 3], Yi Ding[4], Peizeng Jia[5, *], Huaxiang Zhao[1, 2, *]

*** Correspondence to:**

peizengjia@hotmail.com (Peizeng Jia) and huaxiangzhao@xjtu.edu.cn (Huaxiang Zhao)

The Supplemental figures include:

- ✧ **Supplemental Figure 1.** Pretreatment chest radiograph.
- ✧ **Supplemental Figure 2.** Cephalometric superimposition.
- ✧ **Supplemental Figure 3.** Panoramic radiograph post to the teeth extraction surgery.
- ✧ **Supplemental Figure 4.** Facial and intraoral photographs after retention for 10 months.

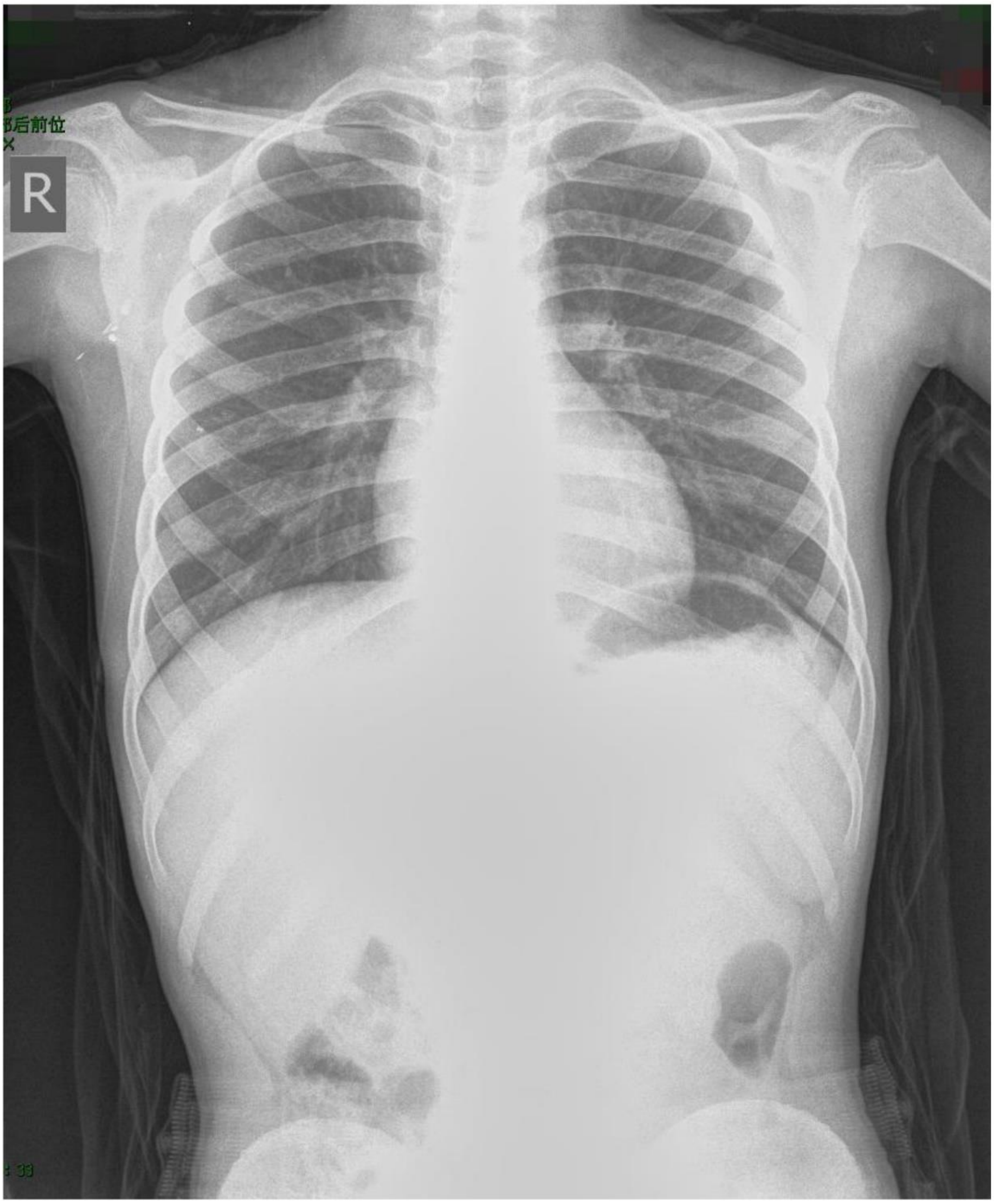

Supplemental Figure 1. Pretreatment chest radiograph.

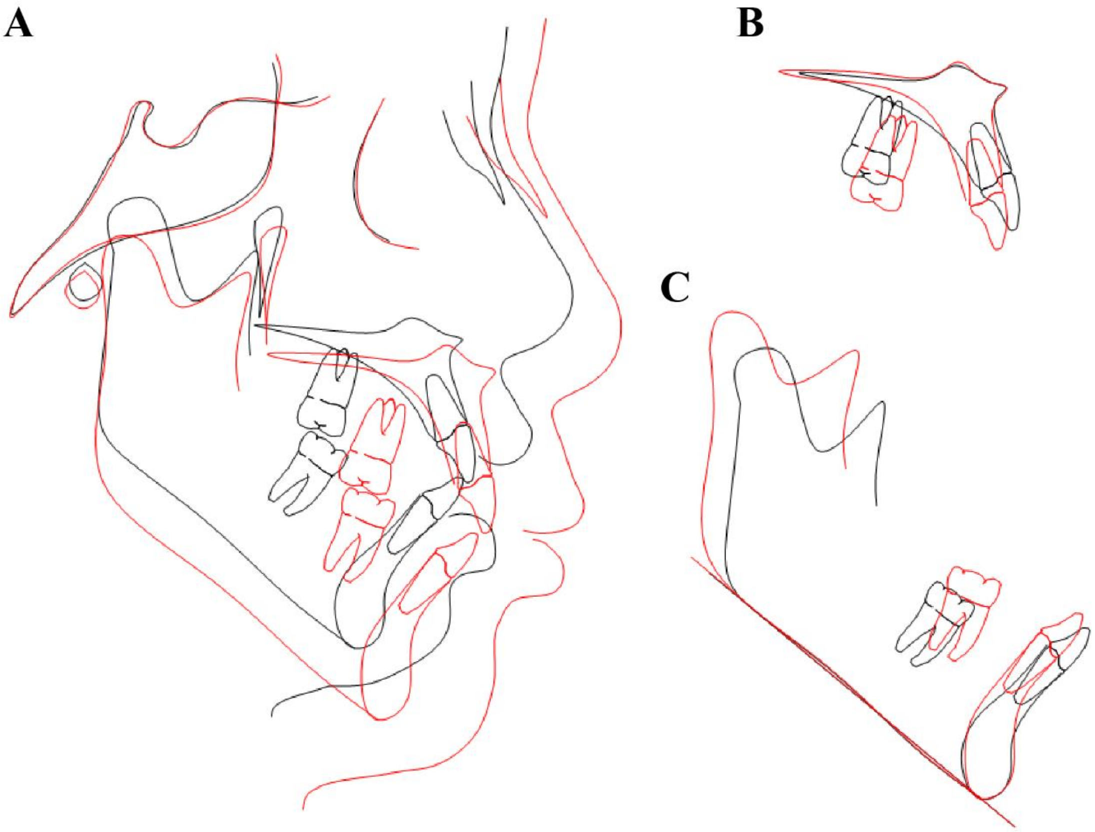

Supplemental Figure 2. Cephalometric superimposition.

(A) The SN plane. **(B)** The maxillary plane. **(C)** The mandibular plane. Black lines indicate the pretreatment cephalometric tracing, while red lines indicate the posttreatment cephalometric tracing.

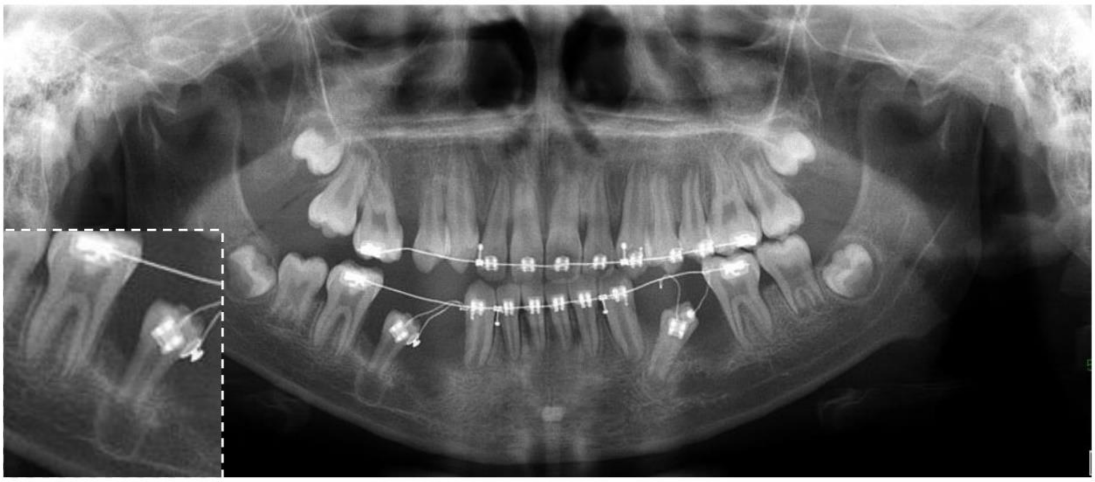

Supplemental Figure 3. Panoramic radiograph post to the teeth extraction surgery. The dashed boxed area is shown at higher magnification. It is noteworthy that no radiographic images of supernumerary teeth were observed mesially to the root of the lower right first molar.

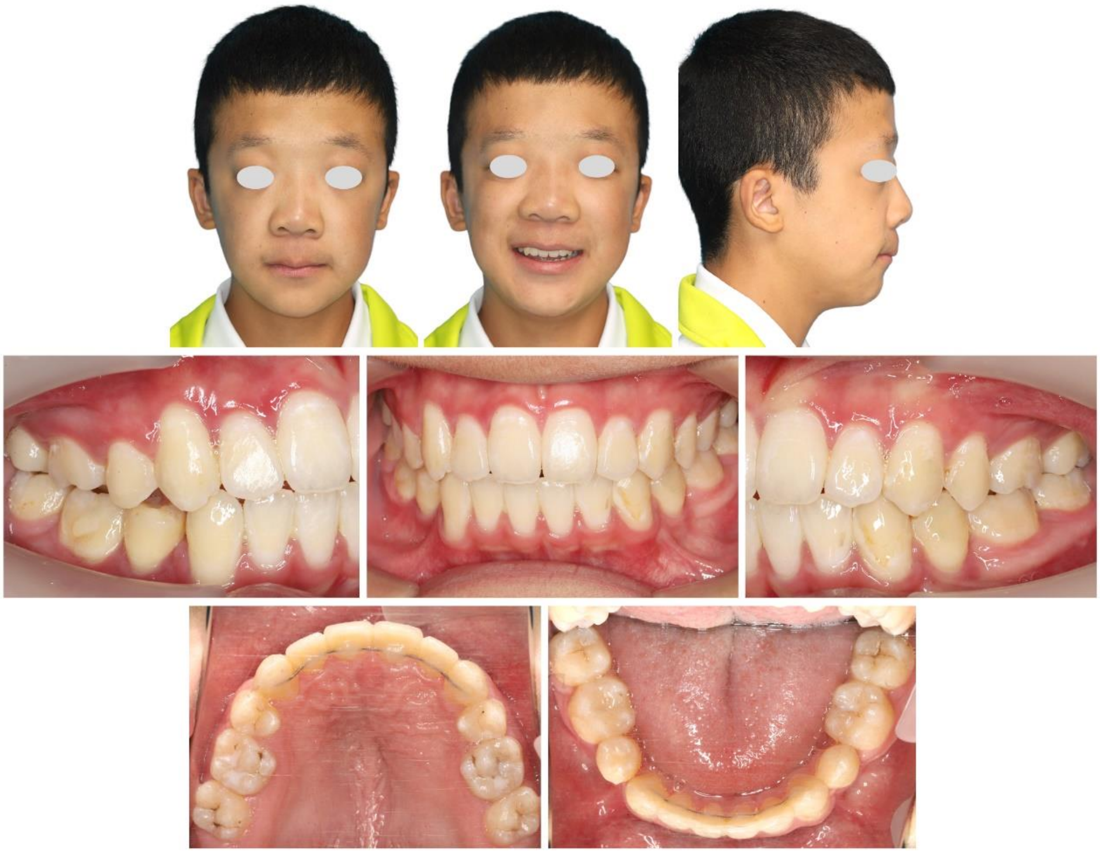

Supplemental Figure 4. Facial and intraoral photographs after retention for 10 months.
